# Epigallocatechin-3-Gallate Enhances the Therapeutic Effects of Leptomycin B on Human Lung Cancer A549 Cells

**DOI:** 10.1155/2015/217304

**Published:** 2015-04-01

**Authors:** Meghan M. Cromie, Weimin Gao

**Affiliations:** Department of Environmental Toxicology, The Institute of Environmental and Human Health, Texas Tech University, Lubbock, TX, USA

## Abstract

Our previous studies have shown Leptomycin B (LMB) is a promising antilung cancer drug. Epigallocatechin-3-gallate (EGCG) has antitumor properties but a debatable clinical application. The objective of this study is to evaluate the combination therapeutic effect of LMB and EGCG and its molecular mechanisms in human lung cancer A549 cells. Increased cytotoxicity was observed in LMB+EGCG-treated cells compared to LMB-treated cells. Elevated ROS was maximized 2 h after treatment, and LMB+EGCG-treated cells had higher ROS levels compared to LMB. N-Acetyl-L-cysteine (NAC) studies confirmed the oxidative role of LMB and/or EGCG treatment. In comparison to the control, CYP3A4, SOD, GPX1, and p21 mRNA expression levels were increased 7.1-, 2.0-, 4.6-, and 13.1-fold in LMB-treated cells, respectively, while survivin was decreased 42.6-fold. Additionally, these increases of CYP3A4, SOD, and GPX1 were significantly reduced, while p21 was significantly increased in LMB+EGCG-treated cells compared to LMB-treated cells. The qRT-PCR results for p21 and survivin were further confirmed by Western blot. Our study first shows that LMB produces ROS and is possibly metabolized by CYP3A4, GPX1, and SOD in A549 cells, and combination treatment of LMB and EGCG augments LMB-induced cytotoxicity through enhanced ROS production and the modulation of drug metabolism and p21/survivin pathways.

## 1. Introduction

Lung cancer is the leading cause of cancer related death in both men and women combined, and in 2014 there were estimated 224,210 new cases and 159,260 deaths. Of these cases, approximately 85% were categorized as non-small cell lung cancer (NSCLC), with squamous cell carcinoma, adenocarcinoma, and large cell carcinoma as the other subtypes [1]. Based on the lack of preventative screening and late detection, lung cancer diagnoses are often associated with a serious prognosis. Unlike other cancers, individuals diagnosed with Stage IA lung cancer have merely a 49% chance of a 5-year observed survival rate, whereas Stage I colon cancer is 92% and nearly 100% for Stages I and II in prostate cancer [1]. Identification and utilization of the most useful therapeutic options for patients diagnosed with NSCLC is of great importance, considering the low survival rates.

Traditionally, therapies directed toward vascular endothelial growth factor (VEGF) and epidermal growth factor receptor (EGFR) antagonists are used in NSCLC treatment [2]. Approximately 40% of patients are in advanced stages of NSCLC when undergoing treatment, so combination therapy is administered in cycles, but often times this does not greatly influence survival rates [3]. Compacted by inefficient therapeutic regimens and low survival rates, identification of possible novel and effective therapeutics in NSCLC is of utmost importance. Leptomycin B (LMB) is classified as a broad-spectrum antitumor antibiotic and it is derived from* Streptomyces* sp. Strain ATS1287 [4]. LMB acts on the chromosomal region maintenance 1 (CRM1) protein that is responsible for the nuclear export of RNA, as well as proteins involved in tumor suppression, apoptosis, and cell cycle progression, from the nucleus to the cytoplasm [5]. Through Michael-type addition reactions, LMB promotes the alkylation of cysteine 528 (Cys528), which ultimately inhibits the construction of the CRM1-cargo-RanGTP export complex responsible for nuclear transport to the cytoplasm [6]. In preliminary* in vitro* and murine* in vivo* testing, LMB was regarded as a promising therapeutic option against a multitude of cancer cell cultures and experimental tumors [7–12]. However, in Phase I clinical trials, LMB demonstrated unusual toxicity in patients resulting in malaise, vomiting, and anorexia, thereby contributing to the cessation of LMB clinical trials [13]. With the encouraging experimental findings* in vivo* and* in vitro*, it is worthwhile to identify possible compounds that could be coupled with LMB to enhance its effects, while decreasing the dose and reducing unwanted side effects.

Green tea is one of the most consumed beverages worldwide and epigallocatechin-3-gallate (EGCG) is the major catechin in green tea, followed by epigallocatechin, epicatechin-3-gallate, and epicatechin. EGCG alone comprises 50–80% of the total catechin content in green tea [14]. At present, research substantiates that green tea polyphenols promote antimutagenic, anticarcinogenic, and antitumor properties, so the role of green tea in chemoprevention and its possible therapeutic effects is of great value [15]. More specifically, EGCG has been found to promote cell cycle arrest [16], inhibit cellular proliferation [17], stimulate the induction of apoptosis [18], inhibit metastasis, and inhibit angiogenesis as well [19]. Green tea demonstrates inhibitory effects in a variety of cancers in animal models including lung cancer, while epidemiological studies involving humans have been inconclusive [20]. To date, no studies have examined the implementation of combination treatment of LMB with EGCG in lung cancer cells. In the present study, we found that LMB coupled with an experimentally relevant EGCG concentration augments the cytotoxic effect of LMB in lung adenocarcinoma A549 cells, possibly through regulating metabolic and p21/survivin pathways.

## 2. Materials and Methods

### 2.1. Chemicals

LMB (LC Labs, Woburn, MA) was dissolved in ethanol (Sigma, St. Louis, MO) to make a 10 *μ*M stock solution and EGCG (≥95%, Sigma, St. Louis, MO) was dissolved in dimethyl sulfoxide (DMSO, Sigma, St. Louis, MO) to make a 50 mM stock solution and stored at −80°C in an amber vial. N-Acetyl-L-cysteine (NAC, Sigma, St. Louis, MO) was dissolved in water to make a 0.5 M stock solution. RPMI-1640 growth medium, penicillin/streptomycin, and fetal bovine serum (FBS) were purchased from Thermo Scientific (Pittsburg, PA). Hyclone (1x) porcine trypsin was purchased from GE Healthcare Life Sciences (Logan, UT). 3-(4,5-Dimethylthiazol-2-yl)-2,5-diphenyltetrazolium bromide (MTT) was purchased from Usb Corporation (CA, USA) and dichlorofluorescein diacetate (DCFDA) was purchased from Sigma (St. Louis, MO). Phosphate buffered saline (1x PBS) was purchased from Gibco Life Technologies (Grand Island, NY). RNeasy Blood and Tissue kits were purchased from QIAGEN (MD, USA). The one-step real time-polymerase chain reaction (RT-PCR) kit with SYBR green was purchased from Bio-Rad (Hercules, CA). The following primers for PCR were purchased from Eurofins (Luxembourg): CYP 1A1/1A2/1B1/3A4, GSTP1, Mn-superoxide dismutase (SOD), glutathione peroxidase 1 (GPX1), p53, p21, survivin, CRM1, and glyceraldehyde 3-phosphate dehydrogenase (GAPDH). Radioimmunoprecipitation assay (RIPA) lysis buffer was purchased from Santa Cruz Biotechnology (Santa Cruz, CA). The primary antibodies, p21 and survivin, were purchased from Santa Cruz Biotechnology. *α*-Tubulin was used as the internal control and was purchased from Cell Signaling (Beverly, MA). Horseradish peroxidase- (HRP-) conjugated donkey anti-rabbit IgG and an enhanced chemiluminescence (ECL) kit were purchased from GE Healthcare (Piscataway, NJ).

### 2.2. Cell Culture

Human lung adenocarcinoma cells, A549, were purchased from the American Type Culture Collection (ATCC). Cell culture was conducted following our previous study [12]. In short, A549 cells were cultured in RPMI-1640 medium supplemented with 5% FBS, 50 U/mL penicillin, and 50 mg/mL streptomycin, and the cells were incubated at 37°C in a humidified incubator containing 95% air and 5% CO_2_. Once the cells reached approximately 80% confluency, they were subcultured or plated using trypsin (0.25%).

### 2.3. MTT Assay

A549 cells were seeded into 96-well plates at a density of 5 × 10^3^ cells per well. Cells were treated with the following concentrations: (1) EGCG alone (0–160 *μ*M) for 24 and 48 h, (2) LMB (0–10 nM) alone for 24 and 48 h, and (3) LMB (0–10 nM) + EGCG (20 *μ*M) cotreatment for 24 and 48 h. The treatment medium was aspirated from the wells and 10 *μ*L of 5 mg/mL of MTT was added to 90 *μ*L of new medium to each well and incubated for 3 h at 37°C. After incubation, the MTT was removed from the wells and the cells were dissolved with 100 *μ*L of 100% DMSO and absorbance was measured at 570 nm (reference wavelength of 630 nm) using a Synergy 4 BioTek microplate reader (BioTek Instruments, Inc., Vermont). This experiment was performed independently in triplicate, with a total of 6 replicates per treatment in each experiment. The percent of cell viability was calculated using the following equation: mean absorbance of test wells/mean absorbance of control wells × 100.

### 2.4. ROS Production

As previously described, DCFDA was used to measure reactive oxygen species (ROS) activity in A549 cells [21]. A549 cells were seeded into 96-well plates at 5 × 10^3^ cells per well. Cells were treated for 30 min, 2, 4, 8, 12, 16, 24, and 48 h in 3 independent experiments. In order to confirm our results that LMB + EGCG treatment promoted the formation of ROS, we further conducted DCFDA assays with the well-studied and commonly used ROS scavenger, NAC [22, 23]. Based on the findings of other studies [24–26] and our preliminary MTT data for NAC, A549 cells were pretreated with 10 mM of NAC for 2 h. After treatment, cells were washed twice with 100 *μ*L 1x PBS and then incubated in 100 *μ*L 1x PBS containing 20 *μ*M (final concentration) DCFDA for 45 min at 37°C. ROS fluorescence was then measured at an excitation of 485 nm and an emission of 535 nm using a Synergy 4 BioTek microplate reader (BioTek Instruments, Inc.). The fluorescence in treated cells at each time point was compared to its respective control.

### 2.5. Isolation of Total RNA

Following the RNeasy plus mini kit manual instructions, total RNA was isolated from 1 × 10^6^ A549 cells treated in 6 well plates for 48 h. Cell lysis buffer was added to each well and scraped, and the genomic DNA was removed from the lysate after homogenization. The RNA was eluted by the addition of 30 *μ*L of RNase-free water. The RNA was stored in the −80°C freezer until needed. The concentration of RNA was measured by a Nanodrop 1000 Spectrophotometer (Thermo Scientific, Waltham, MA) at an OD of 260 nm. RNA samples with OD A260/A280 ratios within 1.9–2.1 were used for qRT-PCR.

### 2.6. Quantitative Real-Time PCR (qRT-PCR)

Total RNA (50 ng) was collected from cells treated for 48 h and amplified using a one-step RT-PCR kit with SYBR green following manufacturer instructions. A CFX96 Touch Real-Time PCR Detection System (Bio-Rad) was used to perform single-step RT-PCR amplifications after reverse transcription. Reverse transcription occurred as follows: 50°C for 15 min, denaturation and reverse transcriptase enzyme inactivation at 95°C for 5 min, followed by 40 cycles each containing 10 sec for denaturation at 95°C and 30 sec for annealing and extension at 60°C. Melt curve analysis was used to determine the specificity of the PCR products, and GAPDH served as the housekeeping gene. The Ct values of the genes of interest were normalized with GAPDH and the ΔΔCt method was utilized to calculate the fold-change in gene expression. Nontemplate controls were included for every experiment. Primer sequences are included in Table 1.

### 2.7. Western Blot

A549 cells were treated in petri dishes for 48 h and protein was collected upon washing with 1x PBS and lysing with RIPA lysis buffer. Protein lysate was sonicated and then centrifuged at 13,000 ×g for 5 min and then the supernatant was collected and stored in the −80°C freezer until needed. A Bio-Rad Bradford protein assay was used to determine protein lysate concentration for each treatment. Boiled and chilled protein lysate (30 *μ*g) containing 10 *μ*L of loading dye was loaded into each gel along with the protein ladder (GE Healthcare) and separated by 12% SDS-polyacrylamide gel electrophoresis (SDS-PAGE). The protein was then transferred to a polyvinylidene fluoride (PVDF) membrane using a semidry transfer system (Trans-Blot Semi-Dry transfer cell, Bio-Rad). The membrane was then incubated in blocking buffer comprised of 3% nonfat dry milk in 1x tris-buffered saline and 0.1% Tween20 (1x TBST) for 1 h. Following blocking, the membrane was placed in the appropriate primary antibody overnight at 4°C. Antibodies were detected using HRP-conjugated donkey anti-rabbit IgG (1 : 1000) and ECL. Visualization of the protein on the membrane occurred upon exposure to X-ray film.

### 2.8. Statistical Analyses

Factorial analysis of variance (ANOVA) was performed to test the effects of LMB and/or EGCG on cell cytotoxicity and ROS production. One-way ANOVA was used to determine the difference in gene expression between groups. All analyses were performed using SPSS 13.0 software and differences at *P* < 0.05 were considered statistically significant.

## 3. Results

### 3.1. EGCG and LMB Cytotoxicity

**Figure 1:**
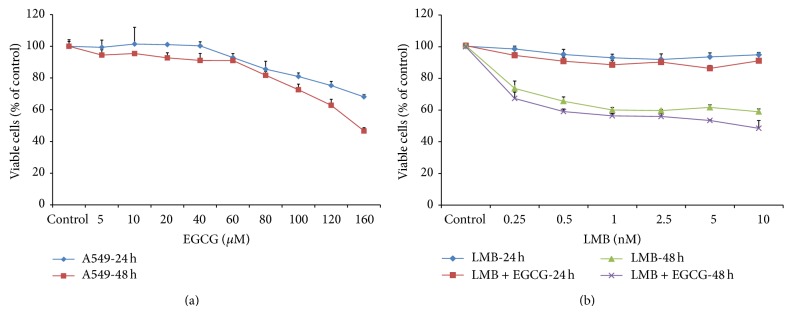
(a) The cytotoxicity of EGCG on A549 cells. Cells were seeded in 96-well plates (5 × 10^3^ cells/well) and treated with EGCG (0–160 *μ*M) for 24 and 48 h. (b) The cytotoxicity of LMB and LMB + EGCG on A549 cells. Cells were treated with LMB (0–10 nM) for 24 and 48 h or LMB (0–10 nM) + EGCG (20 *μ*M) cotreatment for 24 and 48 h. After treatment, 10 *μ*L of 5 mg/mL MTT was added to each well and incubated at 37°C for 3 h. The medium was aspirated and the remaining formazan was solubilized with DMSO and absorbance was measured at 570 nm (reference wavelength of 630 nm). Data are expressed as the percentage of the value as compared to the cells treated with the vehicle control. Data represented as mean ± SE.

The effect of EGCG on A549 cells was determined by MTT assay. After 24 and 48 h of EGCG treatment (5–160 *μ*M), cytotoxicity was induced in a dose-dependent manner (Figure 1(a)). EGCG concentrations at 40 *μ*M or greater significantly induced cytotoxicity in A549 cells when compared to the control (*P* < 0.005), so 20 *μ*M EGCG was used for subsequent combination studies with LMB. Additionally, 20 *μ*M of EGCG is regarded as a low and safe concentration of EGCG for human subjects [27]. A549 cells were exposed to varying concentrations of EGCG (0, 5, 10, and 20 *μ*M) and no changes in mRNA expression of CRM1 were observed upon treatment (data not shown).

In order to determine the effects of individual LMB treatment and cotreatment of LMB with 20 *μ*M of EGCG (LMB + EGCG) in A549 cells, MTT assay was implemented. Based on previous preliminary studies conducted in our lab [11, 12], 0.25–10 nM LMB was used and data were collected at 24 and 48 h.  Figure 1(b) shows the number of viable cells after 24 h of treatment. No remarkable changes in cytotoxicity were observed in cells treated with either LMB or LMB + EGCG for 24 h.  Figure 1(b) also demonstrates reductions in the number of viable cells after 48 h. After 48 h, LMB and/or LMB + EGCG significantly induced cytotoxicity in A549 cells (*P* < 0.001). Moreover, this reduction was more pronounced in LMB + EGCG as compared to LMB alone (*P* < 0.001).

### 3.2. Effects of LMB and EGCG on ROS

**Figure 2:**
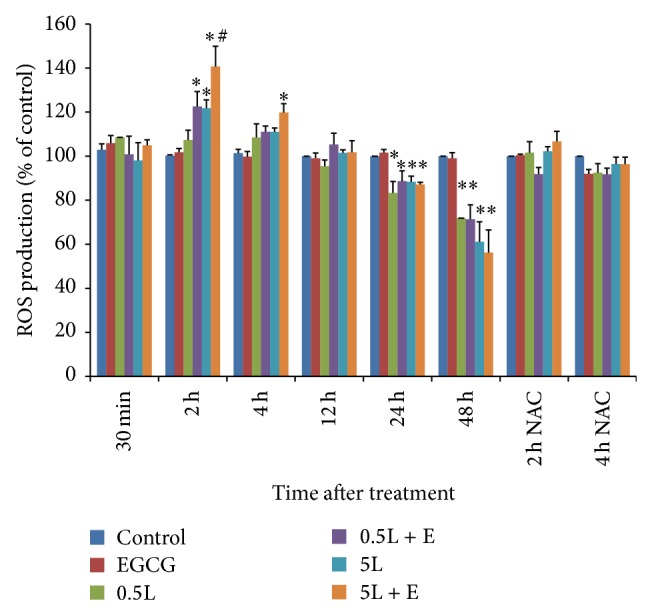
The effects of LMB, LMB + EGCG, or NAC on ROS production. Data are expressed as means ± SE of three replicate experiments. Cells were seeded in 96-well plates (5 × 10^3^ cells/well) and treated for 30 min, 2, 4, 12, 24, and 48 h. A549 cells were also pretreated for 2 h with 10 mM NAC and then treated with LMB and/or EGCG for 2 and 4 h upon NAC removal. ROS formation in cells was detected using DCFDA and the ROS fluorescence was measured at an excitation of 485 nm and an emission of 535 nm. The fluorescence in treated cells at each time point was compared to the respective control. E: 20 *μ*M EGCG; 0.5L: 0.5 nM LMB; 5L: 5 nM LMB; NAC: 10 mM NAC pretreated for 2 h. ^∗^ *P* < 0.05 as compared to control; ^#^ *P* < 0.05 as compared to 5 nM LMB.

Factorial ANOVA analysis showed a significant association between ROS formation and treatment time (*P* < 0.001) and the treatment time and dose interaction (*P* < 0.001) (Figure 2). ROS formation was not altered in cells treated with LMB and/or EGCG for 30 min, 8, 12, and 16 h (data not shown for 8 and 16 h). EGCG by itself did not change ROS formation at any tested time points. The maximum ROS formation was observed 2 h after treatment in 5 nM LMB + EGCG. A significant ROS induction was observed in cells treated with 0.5 nM LMB + EGCG, 5 nM LMB, and 5 nM LMB + EGCG at 2 h as well as 5 nM LMB + EGCG at 4 h (*P* < 0.05, as compared to DMSO control). In addition, ROS formation at 2 h significantly increased in 5 nM LMB + EGCG compared to 5 nM LMB (*P* < 0.05). Significant reduction in ROS occurred in A549 cells treated with LMB or LMB + EGCG for 24 and 48 h (*P* < 0.05, as compared to DMSO control), and this phenomenon was more remarkable in cells after 48-hour treatment compared to 24-hour treatment (*P* < 0.001). Additionally, as shown in Figure 2, cells pretreated with NAC for 2 h significantly decreased ROS formation in A549 cells treated with LMB and/or EGCG for 2 and 4 h (*P* < 0.01), and a significant interaction between treatment groups and NAC was observed (*P* < 0.05).

### 3.3. Effects of LMB and EGCG on Phase I Metabolism

**Figure 3:**
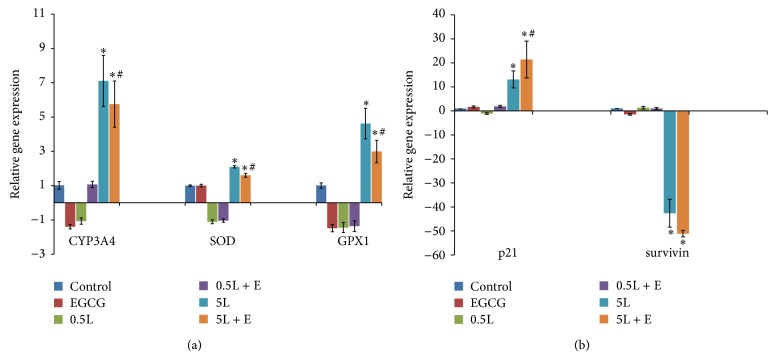
qRT-PCR results of (a) CYP3A4, SOD, and GPX1 gene expression and (b) p21 and survivin gene expression in A549 cells treated for 48 h. Total RNA was collected from the treated cells following the RNeasy kit instructions. Total RNA (50 ng) was then amplified using a one-step RT-PCR kit with SYBR green. The Ct values of the genes of interest were normalized to GAPDH and the fold-change in gene expression was calculated using the ΔΔCt method. Nontemplate controls were included in every experiment. E: 20 *μ*M EGCG; 0.5L: 0.5 nM LMB; 5L: 5 nM LMB; ^∗^ *P* < 0.05 as compared to control; ^#^ *P* < 0.05 as compared to 5 nM LMB.

In order to determine possible metabolic regulation, the following cytochrome P450 enzymes (CYPs) were examined using qRT-PCR: CYP1A1/1B1/1A2/3A4 (CYP1A1, CYP1B1, and CYP1A2 data not shown). A549 cell treatment groups were vehicle control (0.1% DMSO), 20 *μ*M EGCG, 0.5 nM LMB, 0.5 nM LMB + EGCG, 5 nM LMB, and 5 nM LMB + EGCG. Of all the CYPs measured, CYP3A4 was the only metabolic enzyme to express a significant change in gene expression (Figure 3(a)). CYP3A4 expression was significantly upregulated 7.1-fold (*P* < 0.05) and 5.8-fold (*P* < 0.05) in 5 nM LMB and 5 nM LMB + EGCG-treated cells compared to the control, respectively. In addition, CYP3A4 expression significantly decreased in the 5 nM LMB + EGCG-treated cells when compared to the 5 nM LMB alone-treated cells (*P* < 0.05). EGCG-treated cells decreased in CYP3A4 mRNA expression when compared to the control, but the data was not significant.

### 3.4. Effects of LMB and EGCG on Phase II Metabolism

The gene expression levels of possible phase II enzymes, including GSTP1, SOD, and GPX1, were determined by qRT-PCR (Figure 3(a), data not shown for GSTP1). SOD gene expression significantly increased 2.0-fold in cells treated with 5 nM LMB and 1.6-fold in the 5 nM LMB + EGCG-treated cells compared to the control (*P* < 0.05). A significant decrease in SOD expression was observed in the combination treatment as compared to the 5 nM LMB treatment (*P* < 0.05). Similarly, GPX1 gene expression significantly increased 4.6-fold in 5 nM LMB-treated cells and 3.0-fold in 5 nM LMB + EGCG-treated cells (*P* < 0.05) compared to the control. GPX1 gene expression in the combination treated cells was significantly lower than the 5 nM LMB-treated cells (*P* < 0.05).

### 3.5. Effects of LMB and EGCG on Cell Survival

**Figure 4:**
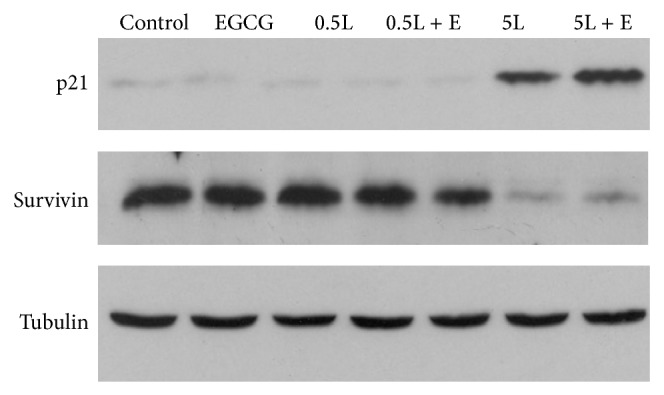
Western blots of p21 and survivin in A549 cells treated for 48 h. Protein was extracted from cells using RIPA lysis buffer. Protein concentration was determined using a Bio-Rad Bradford protein assay. Protein lysate (30 *μ*g) was separated using 12% SDS-PAGE and transferred to a PVDF membrane. After immunoblotting with primary and secondary antibodies, membranes were visualized by chemiluminescence and exposed to X-ray film. E: 20 *μ*M EGCG; 0.5L: 0.5 nM LMB; 5L: 5 nM LMB.

p21 and survivin gene expression were measured by qRT-PCR as well (Figure 3(b)). A549 cells treated with 5 nM LMB and 5 nM LMB + EGCG had a 42.6-fold (*P* < 0.05) and 51.1-fold (*P* < 0.05) decrease in survivin gene expression compared to controls, respectively. Cells treated with 5 nM LMB + EGCG had a greater downregulation of survivin compared to 5 nM LMB alone-treated cells, but it was not significant. p21 gene expression was significantly increased 13.1-fold in 5 nM LMB-treated cells (*P* < 0.05) and 21.4-fold in 5 nM LMB + EGCG-treated cells (*P* < 0.05) compared to the control. A significant increase in p21 gene expression was observed in the 5 nM LMB + EGCG treatment compared to the 5 nM LMB individual treatment (*P* < 0.05). p53 gene expression did not change (data not shown). The results from qRT-PCR for p21 and survivin were further confirmed* via* Western blots (Figure 4).

## 4. Discussion

The results of this study suggest that cell cytotoxicity is enhanced in A549 cells treated with LMB + EGCG compared to LMB alone, especially after 48 h. Furthermore, we demonstrated that ROS levels are greatest after 2 h following treatment. Phase I and II metabolic and oxidative enzymes as well as p21/survivin pathways are potentially responsible for the aforementioned observations. In order to identify an appropriate EGCG concentration to use for this study, an MTT assay was designed to test experimentally relevant concentrations (5–160 *μ*M) after 24 and 48 h. Furthermore, studies conducted in different cell lines have also found that EGCG treatment at a higher concentration for 24–72 h could promote significant increases in cytotoxicity [28, 29]. For instance, MCF-7 breast carcinoma cells exposed to approximately 200 *μ*M of EGCG for 24 h and V79-4 Chinese hamster lung cells exposed to approximately 100 *μ*M of green tea polyphenols for 48 h heeded a considerable decrease in cell viability [28, 30]. EGCG has been found to reduce cell viability in A549 cells through the inhibition of the proapoptotic gene, B-cell lymphoma-extra large (Bcl-xL) [31]. Additionally, Sakamoto et al. found that EGCG suppressed A549 cell growth through the inhibition of VEGF expression and stimulation of endostatin [32]. Our results found that 20 *μ*M EGCG resulted in no-observed cytotoxicity; therefore, all combination studies used this concentration.

Previous LMB studies conducted by our lab supported test concentrations of 0.25–10 nM LMB for 24 and 48 h LMB treatment [11, 12]. At 48 h, a marked reduction in cell cytotoxicity was observed in LMB and LMB + EGCG-treated A549 cells. Additionally, a previous study conducted by our lab found LMB-induced apoptosis and G2/M arrest in A549 cells, when compared to the control, using similar doses as were selected in the present study [11]. Cells dosed with LMB + EGCG had a greater overall cytotoxicity compared to the LMB alone-treated cells. These results were further corroborated by studies examining classic chemotherapy drugs in various cancer cell lines. A comprehensive review by Suganuma et al. found similar results in that EGCG combined with 5-fluorouracil [20], taxol [33], doxorubicin [34], gefitinib [35], or erlotinib [36] resulted in a synergistic growth inhibition in various cancers [37]. Cisplatin, a commonly employed platinum-based therapeutic in NSCLC treatment, is also highly effective, but oftentimes lung cancer cells become chemoresistant. A study conducted by Kim and Lee found that EGCG induced cytotoxicity in cisplatin-resistant A549 cells through the downregulation of cell survival proliferation genes, Axl and Tyro 3 [38]. Due to the results of these findings, it appears that LMB and EGCG cotreatment have a concerted effect on cellular growth inhibition in A549 cells and even have the potential to be effective in resistant cell lines.

The cytotoxic effects of EGCG were also tested in A549 cells with CRM1 stable knockdown (A549_CRM1−_), a cell line generated by our group using a short hairpin (shRNA) and pSilencer 4.1-CMV plasmid by G418 selection [39]. EGCG resulted in a greater cytotoxic effect in A549_CRM1−_ cells than wild type A549 cells (data not shown). A recent study demonstrated that curcumin, a major component of turmeric, targeted CRM1 and more specifically Cys528 and promoted the nuclear retention of FOXO1, which ultimately impacts cell cycle cascades [40]. Since EGCG, which differs from curcumin, did not change CRM1 expression, the observed synergistic cytotoxic effect of EGCG on A549_CRM1−_ could occur through an alternative but compensatory pathway(s). Future studies are needed to understand these additional mechanisms. Alternatively, due to the toxic nature of LMB observed in Phase I clinical trials, other CRM1 inhibitors have been developed and are undergoing testing. CBS9106 is a reversible CRM1 inhibitor that exhibited antitumor activity against* in vivo* and* in vitro* multiple myeloma cells [41]. Mutka et al. developed novel nuclear export inhibitors similar to LMB, but with protracted nuclear export and apoptosis in human papilloma squamous cell carcinoma, human colon carcinoma, human neuroblastoma, and the aforementioned cell lines xenografted in athymic mice [42]. The most promising CRM1 inhibitor for NSCLC appears to be KPT-185. Wang et al. found KPT-185 to exert significant cytotoxicity in 6 NSCLC cell lines, including drug resistant cell lines. In addition to* in vivo* studies, KPT-185 greatly reduced tumor size in mouse tumor xenografts [43]. Although these new CRM1 inhibitors appear to be promising based on preliminary studies, they too could be challenged when administered to human subjects, thereby emphasizing the importance of alternative therapeutic options like the one discussed in this study.

Upon exposure to individual and combination treatment, ROS levels were measured at 30 min to 48 h in A549 cells. At 2 h, ROS levels were at their greatest, with the lowest ROS amounts found at 48 h. The decline in ROS levels over time is possibly attributable to overall treatment killing of the cells, thereby resulting in reduced ROS levels. For instance, although the detected ROS level in the treated cells at 48 h was low, the ROS produced by the surviving cells after 48 h treatment could be artificially decreased due to treatment-related cytotoxicity. Therefore, the presence of ROS prior to the 24 h time point elicits cellular pathways that ultimately promote apoptosis in the cells at 48 h. In this study, it appeared that LMB + EGCG combination treatment promoted greater ROS levels than LMB treatment alone. This data suggests that EGCG is not acting as an antioxidant, but it is inducing oxidative stress in the A549 cells. The most recent literature concerning EGCG and ROS formation is equivocal, with many studies reporting conflicting evidence [44]. A study by Benzie and Szeto found a direct positive correlation between green tea polyphenol content and antioxidant activity using the ferric reducing/antioxidant power assay, and they also found that green tea possessed the greatest antioxidative activity when compared to other tea varieties [45]. Green tea extract and EGCG were both found to defend against oxidative stress in normal/malignant human bladder cells [46]. Conversely, Nakazato et al. demonstrated an increase in ROS during EGCG-induced apoptosis in retinoic acid-resistant acute promyelocytic leukemia cells [47]. Similarly, Li et al. found that ROS levels increased during EGCG-induced apoptosis in human hepatoma cells [48]. Additionally, in terms of LMB, Jang et al. showed that LMB treatment in U937 leukemia cells did not result in ROS formation and was not attributable to LMB-induced apoptosis [49]. Little evidence is available with regard to the possible oxidative damage induced by LMB treatment, but our results indicate that LMB could potentially contribute to ROS levels observed in this system. It also appears that, upon initial treatment, ROS levels are greatest in cells cotreated with EGCG, thereby supporting the classification of EGCG as a prooxidant. Finally, our data using NAC pretreatment in A549 cells showed a significant decrease in ROS induction and these findings further support that LMB and/or EGCG combination treatment contributes to the observed ROS formation.

Metabolism of LMB individual and LMB + EGCG combination treatments was determined by testing Phase I enzymes (CYP1A1/1A2/1B1/3A4) using qRT-PCR. The mRNA level of CYP3A4 was the only Phase I enzyme to exhibit a change in gene expression upon treatment. CYP3A4 gene levels were greatest at 5 nM LMB, followed by 5 nM LMB + EGCG, suggesting that CYP3A4 is inducible and involved in LMB metabolic pathways. CYP3A4 is an important Phase I enzyme that is responsible for the metabolism of approximately half of prescribed pharmaceuticals [50]; for instance, chemotherapeutics are common substrates of CYP3A4 [51]. Based on our qRT-PCR results, CYP3A4 could play an important role in LMB metabolism and EGCG by itself inhibited, but not significantly, CYP3A4 induction. Nakamura et al. found EGCG to metabolically inhibit CYP3A4 upon treatment [52]. These results might explain the finding regarding the decrease of CYP3A4 in LMB + EGCG combination treatment compared to LMB treatment, which could reduce the LMB Phase I metabolism by EGCG to maintain LMB efficacy and further increase cell cytotoxicity, as observed. Due to the inhibition of CYP3A4 by EGCG, which can be observed in the EGCG alone and the 5 nM LMB + EGCG cotreatments, it is likely that the Phase I metabolism of LMB is delayed. Ultimately, this inhibition promotes elevated LMB concentrations to persist and exert further cytotoxic effects to A549 cells.

**Figure 5:**
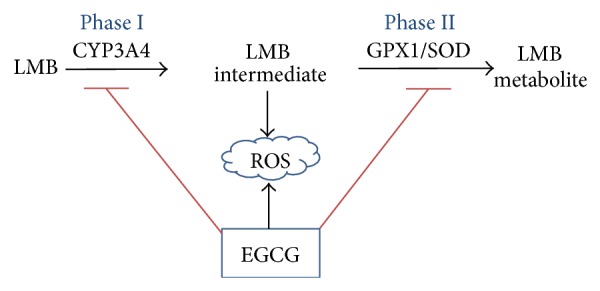
Hypothesis of EGCG on LMB metabolisms. LMB is possibly metabolized by the Phase I enzyme, CYP3A4, followed by Phase II enzymes. EGCG contributes to the formation of ROS, as well as inhibiting Phase I and II enzymes in A549 cells exposed to LMB + EGCG. → induction; ⊥ inhibition.

A similar trend was observed in the Phase II enzymes, SOD and GPX1, with 5 nM LMB having the greatest gene expression, followed by 5 nM LMB + EGCG. It is believed that the upregulation of these antioxidant defense enzymes was observed in response to the enhanced ROS levels following treatment, as well as possible Phase II metabolism of LMB. Both SOD and GPX1 are important to cells because they convert ROS to oxygen and water in order to eliminate oxidative damage in the cell [53]. In accordance with our findings, EGCG displayed prooxidant behaviors in human lung cancer H1299 cells by inducing ROS formation, and SOD was responsible for quenching the ROS formed as a product of EGCG exposure [27]. Additionally, Song et al. found catalase and SOD introduction to eliminate ROS formation in Jurkat and 293T cells [54]. EGCG has been shown to downregulate GPX1 in human hepatocellular carcinoma BEL7402/5-FU cells [55]. Conversely, green tea polyphenols administered to the drinking water of female SKH-1 hairless mice promoted the induction of GPX1 in lung tissues of mice [56]. In another study examining immune regulation conducted by Liu et al., EGCG helped to restore SOD levels in concanavalin A-challenged mice [57]. EGCG was also found to restore SOD and GPX1 in free fatty acid-induced peripheral insulin resistant Wistar rats [48]. Altogether, these controversial findings regarding EGCG on SOD and GPX1 expression need to be further investigated. Nevertheless, little data regarding the metabolism of LMB is currently available and this study offers new insight into the possible metabolic pathways involved in LMB and EGCG treatment of A549 cells (Figure 5).

Next, p21 and survivin mRNA were measured using qRT-PCR. p21 expression was greatest at 5 nM LMB + EGCG, followed by 5 nM LMB alone. Survivin expression decreased with 5 nM LMB + EGCG decreasing the most, followed by 5 nM LMB alone-treated cells. Our previous studies demonstrated that LMB by itself promotes p21 upregulation and survivin downregulation in A549 cells [11, 12]. Similar to the present findings, Fujiki et al. utilized a human cancer cDNA array and found that the chemotherapeutic drug, sulindac, and EGCG cotreatment upregulated p21 expression as well [58]. In lung cancer, p21 is an important inhibitor of cyclin-dependent kinases (CDKs) and it has been shown to be activated* via* p53-dependent [59] and p53-independent [60] pathways. Shoji et al. found that, in NSCLC, p21 serves as a useful prognostic factor in patients since it was measured in 51.5% of the patients tested and the five-year survival rate of those patients was 73.8%, which was significantly greater than the p21-negative patients [61]. The findings of this study emphasize the importance of p21 as a useful biomarker or determinant of a good prognosis, which possibly explains the beneficial effects of LMB and EGCG cotreatment in A549 cells in the present study. Due to the lack of alterations in p53 mRNA expression and p-p53 (Ser15) protein expression (data not shown), it could be deduced that p21 might act through p53-independent pathways in this study. Interestingly, LMB + EGCG combination treatment resulted in a synergistic effect on p21.

Survivin is a member of the inhibitor of apoptosis (IAP) family and it is often overexpressed in NSCLC, which is indicative of a poor prognosis in patients [62]. Cancer cells upregulate and sequester survivin in the cytoplasm where it maintains its antiapoptotic function. The nuclear localization of survivin ultimately enhances the sensitivity of cancer cells to chemotherapeutics [63]. Since LMB inhibits CRM1 nuclear-cytoplasmic export, survivin levels decreased, as expected. Additionally, studies using NUGC-3 gastric cancer cells and MCF-7 breast cancer cells both found that EGCG treatment resulted in significant reductions in survivin expression [64, 65]. A study conducted by Hossain et al. confirmed that survivin is highly expressed in malignant neuroblastoma SK-N-BE2 and SH-SY5Y cells, and upon silencing of survivin, EGCG had a greater anticancer capacity, further suggesting the importance of survivin inhibition in cancer [66]. Based on the findings in the present study, the increased p21 expression prompts a greater inhibition of CDKs which are crucial to cell cycle signaling and transcription, while decreased survivin expression prevents the inhibition of apoptosis. Ultimately, the decreased p21 but increased survivin expression levels could contribute to the observed cytotoxicity in A549 cells upon 5 nM LMB and 5 nM LMB + EGCG treatment.

Overall, this study offers new insight into the metabolism of LMB and cotreatment of A549 cells with LMB and EGCG. The data reveals the enhanced cytotoxicity observed during cotreatment. ROS levels attained maximal concentrations at the initial onset of treatment (2 h), in which EGCG acts as a prooxidant, but slowly declined after LMB and EGCG began to elicit their cytotoxic effects. LMB Phase I metabolism appeared to be mediated through CYP3A4, and Phase II oxidative metabolism was facilitated by both SOD and GPX1 enzymes. Finally, EGCG enhanced LMB cytotoxicity appeared to occur through inhibition of these drug metabolism enzymes, upregulation of p21, and downregulation of survivin mRNA and protein expression. Additional studies, such as mouse tumor xenograft studies, could provide important information to help us gain a better understanding revolving the use of the polyphenol, EGCG, in combination with LMB treatment in NSCLC.

## Figures and Tables

**Table 1:** Forward and reverse primers of genes used in qRT-PCR.

Primer	Forward primer (5′ to 3′)	Reverse primer (5′ to 3′)
GAPDH	GGTGGTCTCCTCTGACTTCAACA	GTTGCTGTAGCCAAATTCGTTGT
CRM1	GGAACCAGTGCGAAGGAATA	TTTCGCTGGTCCTACTTGCT
CYP1A1	TGGTCTCCCTTCTCTACACTCTTGT	ATTTTCCCTATTACATTAAATCAATGGTTCT
CYP1A2	AACAAGGGACACAACGCTGAAT	GGAAGAGAAACAAGGGCTGAGT
CYP1B1	CACTGCCAACACCTCTGTCTT	CAAGGAGCTCCATGGACTCT
CYP3A4	CTTCATCCAATGGACTGCATAAAT	TCCCAAGTATAACACTCTACACAGACAA
SOD	GCTGACGGCTGCATCTGTT	CCTGATTTGGACAAGCAGCAA
GPX1	TATCGAGAATGTGGCGTCCC	TCTTGGCGTTCTCCTGATGC
GSTP1	GCCCTACACCGTGGTCTATT	GGTCTCCGTCCTGGAACTTG
p21	GACACCACTGGAGGGTGACT	CAGGTCCACATGGTCTTCCT
Survivin	AGCCAGATGACGACCCCATT	GCAACCGGCCGAATGCTTTT
p53	GGCCCACTTCACCGTACTAA	GTGGTTTCAAGGCCAGATGT

## References

[1] (2014). *Cancer Facts & Figures*.

[2] Pal S. K., Figlin R. A., Reckamp K. (2010). Targeted therapies for non-small cell lung cancer: an evolving landscape. *Molecular Cancer Therapeutics*.

[3] Dempke W. C. M., Suto T., Reck M. (2010). Targeted therapies for non-small cell lung cancer. *Lung Cancer*.

[4] Yoshida M., Nishikawa M., Nishi K., Abe K., Horinouchi S., Beppu T. (1990). Effects of leptomycin B on the cell cycle of fibroblasts and fission yeast cells. *Experimental Cell Research*.

[5] Nguyen K. T., Holloway M. P., Altura R. A. (2012). The CRM1 nuclear export protein in normal development and disease. *International Journal of Biochemistry and Molecular Biology*.

[6] Kudo N., Matsumori N., Taoka H. (1999). Leptomycin B inactivates CRM1/exportin 1 by covalent modification at a cysteine residue in the central conserved region. *Proceedings of the National Academy of Sciences of the United States of America*.

[7] Komiyama K., Okada K., Tomisaka S., Umezawa I., Hamamoto T., Beppu T. (1985). Antitumor activity of leptomycin B. *Journal of Antibiotics*.

[8] Leopold W. R., Shillis J. L., Mertus A. E., Nelson J. M., Roberts B. J., Jackson R. C. (1984). Anticancer activity of the structurally novel antibiotic CI-920 and its analogues. *Cancer Research*.

[9] Roberts B. J., Hamelehle K. L., Sebolt J. S., Leopold W. R. (1986). In vivo and in vitro anticancer activity of the structurally novel and highly potent antibiotic CI-940 and its hydroxy analog (PD 114,721). *Cancer Chemotherapy And Pharmacology*.

[10] Tunac J. B., Graham B. D., Dobson W. E., Lenzini M. D. (1985). Novel antitumor antibiotics, CI-940 (PD 114,720) and PD 114,721. Taxonomy, fermentation and biological activity. *Journal of Antibiotics*.

[11] Lu C., Shao C., Cobos E., Singh K. P., Gao W. (2012). Chemotherapeutic sensitization of leptomycin B resistant lung cancer cells by pretreatment with doxorubicin. *PLoS ONE*.

[12] Shao C., Lu C., Chen L., Koty P. P., Cobos E., Gao W. (2011). p53-dependent anticancer effects of leptomycin B on lung adenocarcinoma. *Cancer Chemotherapy and Pharmacology*.

[13] Newlands E. S., Rustin G. J. S., Brampton M. H. (1996). Phase I trial of elactocin. *British Journal of Cancer*.

[14] Feng W. Y. (2006). Metabolism of green tea catechins: an overview. *Current Drug Metabolism*.

[15] Brown M. D. (1999). Green tea (Camellia sinensis) extract and its possible role in the prevention of cancer. *Alternative Medicine Review*.

[16] Kim C.-H., Moon S.-K. (2005). Epigallocatechin-3-gallate causes the p21/WAF1-mediated G_1_-phase arrest of cell cycle and inhibits matrix metalloproteinase-9 expression in TNF-*α*-induced vascular smooth muscle cells. *Archives of Biochemistry and Biophysics*.

[17] Thangapazham R. L., Singh A. K., Sharma A., Warren J., Gaddipati J. P., Maheshwari R. K. (2007). Green tea polyphenols and its constituent epigallocatechin gallate inhibits proliferation of human breast cancer cells in vitro and in vivo. *Cancer Letters*.

[18] Ahn W. S., Huh S. W., Bae S.-M. (2003). A major constituent of green tea, EGCG, inhibits the growth of a human cervical cancer cell line, CaSki cells, through apoptosis, G_1_ arrest, and regulation of gene expression. *DNA and Cell Biology*.

[19] Shankar S., Ganapathy S., Hingorani S. R., Srivastava R. K. (2008). EGCG inhibits growth, invasion, angiogenesis and metastasis of pancreatic cancer. *Frontiers in Bioscience*.

[20] Yang X.-W., Wang X.-L., Cao L.-Q. (2012). Green tea polyphenol epigallocatechin-3-gallate enhances 5-fluorouracil-induced cell growth inhibition of hepatocellular carcinoma cells. *Hepatology Research*.

[21] Zhu W., Cromie M. M., Cai Q., Lv T., Singh K., Gao W. (2014). Curcumin and vitamin E protect against adverse effects of benzo[a]pyrene in lung epithelial cells. *PLoS ONE*.

[22] Yang K. M., Kim B. M., Park J.-B. (2014). *ω*-Hydroxyundec-9-enoic acid induces apoptosis through ROS-mediated endoplasmic reticulum stress in non-small cell lung cancer cells. *Biochemical and Biophysical Research Communications*.

[23] You B. R., Park W. H. (2014). Zebularine inhibits the growth of A549 lung cancer cells via cell cycle arrest and apoptosis. *Molecular Carcinogenesis*.

[24] Ganguli A., Choudhury D., Datta S., Bhattacharya S., Chakrabarti G. (2014). Inhibition of autophagy by chloroquine potentiates synergistically anti-cancer property of artemisinin by promoting ROS dependent apoptosis. *Biochimie*.

[25] Guo J., Wu G., Bao J., Hao W., Lu J., Chen X. (2014). Cucurbitacin B induced ATM-mediated DNA damage causes G2/M cell cycle arrest in a ROS-dependent manner. *PLoS ONE*.

[26] Li L., Qiu P., Chen B. (2014). Reactive oxygen species contribute to arsenic-induced EZH2 phosphorylation in human bronchial epithelial cells and lung cancer cells. *Toxicology and Applied Pharmacology*.

[27] Li G.-X., Chen Y.-K., Hou Z. (2010). Pro-oxidative activities and dose-response relationship of (-)-epigallocatechin-3-gallate in the inhibition of lung cancer cell growth: a comparative study *in vivo* and *in vitro*. *Carcinogenesis*.

[28] Hoon H. D., Jeong J. H., Kim H. J. (2009). Anti-proliferative and apoptosis induction activity of green tea polyphenols on human promyelocytic leukemia HL-60 cells. *Anticancer Research*.

[29] Inoue H., Maeda-Yamamoto M., Nesumi A., Murakami A. (2012). Delphinidin-3-O-galactoside protects mouse hepatocytes from (-)-epigallocatechin-3-gallate-induced cytotoxicity via up-regulation of heme oxygenase-1 and heat shock protein 70. *Nutrition Research*.

[30] Farabegoli F., Barbi C., Lambertini E., Piva R. (2007). (−)-Epigallocatechin-3-gallate downregulates estrogen receptor alpha function in MCF-7 breast carcinoma cells. *Cancer Detection and Prevention*.

[31] Sonoda J. I., Ikeda R., Baba Y. (2014). Green tea catechin, epigallocatechin-3-gallate, attenuates the cell viability of human non-small-cell lung cancer A549 cells via reducing Bcl-xL expression. *Experimental and Therapeutic Medicine*.

[32] Sakamoto Y., Terashita N., Muraguchi T., Fukusato T., Kubota S. (2013). Effects of epigallocatechin-3-gallate (EGCG) on a549 lung cancer tumor growth and angiogenesis. *Bioscience, Biotechnology and Biochemistry*.

[33] Masuda M., Suzui M., Lim J. T. E., Weinstein I. B. (2003). Epigallocatechin-3-gallate inhibits activation of HER-2/neu and downstream signaling pathways in human head and neck and breast carcinoma cells. *Clinical Cancer Research*.

[34] Liang G., Tang A., Lin X. (2010). Green tea catechins augment the antitumor activity of doxorubicin in an in vivo mouse model for chemoresistant liver cancer. *International Journal of Oncology*.

[35] Chang C.-M., Chang P.-Y., Tu M.-G. (2012). Epigallocatechin gallate sensitizes CAL-27 human oral squamous cell carcinoma cells to the anti-metastatic effects of gefitinib (Iressa) via synergistic suppression of epidermal growth factor receptor and matrix metalloproteinase-2. *Oncology Reports*.

[36] Liang Y., Lin-shiau S., Chen C., Lin J. (1997). Suppression of extracellular signals and cell proliferation through EGF receptor binding by (−)-epigallocatechin gallate in human A431 epidermoid carcinoma cells. *Journal of Cellular Biochemistry*.

[37] Suganuma M., Saha A., Fujiki H. (2011). New cancer treatment strategy using combination of green tea catechins and anticancer drugs. *Cancer Science*.

[38] Kim K.-C., Lee C. (2014). Reversal of cisplatin resistance by epigallocatechin gallate is mediated by downregulation of Axl and Tyro 3 expression in human lung cancer cells. *The Korean Journal of Physiology & Pharmacology*.

[39] Gao W., Lu C., Chen L., Keohavong P. (2015). Overexpression of CRM1: a characteristic feature in a transformed phenotype of lung carcinogenesis and a molecular target for lung cancer adjuvant therapy. *Journal of Thoracic Oncology*.

[40] Niu M., Wu S., Mao L., Yang Y. (2013). CRM1 is a cellular target of curcumin: new insights for the myriad of biological effects of an ancient spice. *Traffic*.

[41] Sakakibara K., Saito N., Sato T. (2011). CBS9106 is a novel reversible oral CRM1 inhibitor with CRM1 degrading activity. *Blood*.

[42] Mutka S. C., Yang W. Q., Dong S. D. (2009). Identification of nuclear export inhibitors with potent anticancer activity in vivo. *Cancer Research*.

[43] Wang S., Han X., Wang J., Yao J., Shi Y. (2014). Antitumor effects of a novel chromosome region maintenance 1 (CRM1) inhibitor on non-small cell lung cancer cells in vitro and in mouse tumor xenografts. *PLoS ONE*.

[44] Forester S. C., Lambert J. D. (2011). The role of antioxidant versus pro-oxidant effects of green tea polyphenols in cancer prevention. *Molecular Nutrition and Food Research*.

[45] Benzie I. F. F., Szeto Y. T. (1999). Total antioxidant capacity of teas by the ferric reducing/antioxidant power assay. *Journal of Agricultural and Food Chemistry*.

[46] Coyle C. H., Philips B. J., Morrisroe S. N., Chancellor M. B., Yoshimura N. (2008). Antioxidant effects of green tea and its polyphenols on bladder cells. *Life Sciences*.

[47] Nakazato T., Ito K., Miyakawa Y. (2005). Catechin, a green tea component, rapidly induces apoptosis of myeloid leukemic cells via modulation of reactive oxygen species production in vitro and inhibits tumor growth in vivo. *Haematologica*.

[48] Li Y., Zhao S., Zhang W. (2011). Epigallocatechin-3-O-gallate (EGCG) attenuates FFAs-induced peripheral insulin resistance through AMPK pathway and insulin signaling pathway *in vivo*. *Diabetes Research and Clinical Practice*.

[49] Jang B.-C., Muñoz-Najar U., Paik J.-H., Claffey K., Yoshida M., Hla T. (2003). Leptomycin B, an inhibitor of the nuclear export receptor CRM1, inhibits COX-2 expression. *Journal of Biological Chemistry*.

[50] Plant N. J., Gibson G. G. (2003). Evaluation of the toxicological relevance of CYP3A4 induction. *Current Opinion in Drug Discovery & Development*.

[51] Tian D., Hu Z. (2014). CYP3A4-mediated pharmacokinetic interactions in cancer therapy. *Current Drug Metabolism*.

[52] Nakamura T., Asada E., Nagata Y., Kanazawa H. (2003). Effect of metabolic inhibition against CYP3A4 by catechins in bottled green tea drinks. *Bunseki Kagaku*.

[53] Yuzhalin A. E., Kutikhin A. G. (2012). Inherited variations in the SOD and GPX gene families and cancer risk. *Free Radical Research*.

[54] Song S., Huang Y. W., Tian Y., Wang X. J., Sheng J. (2014). Mechanism of action of (–)-epigallocatechin-3-gallate: auto-oxidation-dependent activation of extracellular signal-regulated kinase 1/2 in Jurkat cells. *Chinese Journal of Natural Medicines*.

[55] Tang H. H., Zhou M., Liang G. (2008). Impact of epigallocatechin gallate on gene expression profiles of human hepatocellular carcinoma cell lines BEL7404/ADM and BEL7402/5-FU. *Ai Zheng*.

[56] Khan S. G., Katiyar S. K., Agarwal R., Mukhtar H. (1992). Enhancement of antioxidant and phase II enzymes by oral feeding of green tea polyphenols in drinking water to SKH-1 hairless mice: possible role in cancer chemoprevention. *Cancer Research*.

[57] Liu D., Zhang X., Jiang L., Guo Y., Zheng C. (2014). Epigallocatechin-3-gallate (EGCG) attenuates concanavalin A-induced hepatic injury in mice. *Acta Histochemica*.

[58] Fujiki H., Suganuma M., Kurusu M. (2003). New TNF-*α* releasing inhibitors as cancer preventive agents from traditional herbal medicine and combination cancer prevention study with EGCG and sulindac or tamoxifen. *Mutation Research/Fundamental and Molecular Mechanisms of Mutagenesis*.

[59] Xiong Y., Hannon G. J., Zhang H., Casso D., Kobayashi R., Beach D. (1993). p21 is a universal inhibitor of cyclin kinases. *Nature*.

[60] Marchetti A., Doglioni C., Barbareschi M. (1996). P21 RNA and protein expression in non-small cell lung carcinomas: evidence of p53-independent expression and association with tumoral differentiation. *Oncogene*.

[61] Shoji T., Tanaka F., Takata T. (2002). Clinical significance of p21 expression in non-small-cell lung cancer. *Journal of Clinical Oncology*.

[62] Sasaki T., Lopes M. B. S., Hankins G. R., Helm G. A. (2002). Expression of survivin, an inhibitor of apoptosis protein, in tumors of the nervous system. *Acta Neuropathologica*.

[63] Chan K. S., Wong C. H., Huang Y. F., Li H. Y. (2010). Survivin withdrawal by nuclear export failure as a physiological switch to commit cells to apoptosis. *Cell Death and Disease*.

[64] Onoda C., Kuribayashi K., Nirasawa S. (2011). (-)-Epigallocatechin-3-gallate induces apoptosis in gastric cancer cell lines by down-regulating survivin expression. *International Journal of Oncology*.

[65] Tang Y., Zhao D. Y., Elliott S. (2007). Epigallocatechin-3 gallate induces growth inhibition and apoptosis in human breast cancer cells through survivin suppression. *International Journal of Oncology*.

[66] Hossain M. M., Banik N. L., Ray S. K. (2012). Survivin knockdown increased anti-cancer effects of (−)-epigallocatechin-3-gallate in human malignant neuroblastoma SK-N-BE2 and SH-SY5Y cells. *Experimental Cell Research*.

